# Evaluating the Clinical Equivalence of Truwax® and Ethicon® Bone Waxes for Sternal Wound Hemostasis: A Prospective Randomized Study

**DOI:** 10.7759/cureus.55141

**Published:** 2024-02-28

**Authors:** Ravi S Shetty, Neeraj Prakash, Vinay Krishna, Rakesh K Verma, Guru P Patel, Ashok Moharana, Deepak Siddabasavaiah

**Affiliations:** 1 Cardiothoracic Surgery, Mathikere Sampangi (MS) Ramaiah Medical College and Hospitals, Bengaluru, IND; 2 Cardiothoracic and Vascular Surgery, Laxmipat Singhania (LPS) Institute of Cardiology and Cardiac Surgery, Ganesh Shankar Vidyarthi Memorial (GSVM) Medical College, Kanpur, IND; 3 Clinical Affairs, Healthium Medtech Limited, Bengaluru, IND

**Keywords:** sternal wound hemostasis, sternal dehiscence, mortality, median sternotomy, bone wax

## Abstract

Background: Incidence of sternal dehiscence, wound infection, and mortality are prevalent following sternotomy. Bone wax is widely used over the sternal edges for augmenting hemostasis. This study evaluated the clinical equivalence of Truwax^®^ (Healthium Medtech Limited, Bengaluru, India) with Ethicon^®^ (Johnson & Johnson, New Brunswick, New Jersey, United States) bone wax for sternal wound hemostasis in subjects undergoing surgical procedures by sternotomy.

Methods: The primary endpoint of this prospective (May 2022-April 2023), parallel-group, two-arm, randomized, single-blind, multicenter study was to evaluate the proportion of subjects having sternal dehiscence within 26 weeks of median sternotomy closure. Secondary endpoints assessed the average time to hemostasis on sternum sides, bone wax properties, number of dressing changes, sternal bone instability (clinically/chest radiography), pain, perioperative/postoperative complications, blood and blood products used, duration of intensive care unit (ICU)/hospital stay, reoperations, time taken to return back to work and normal day-to-day activities, subject satisfaction and quality of life (QoL), and adverse events. A probability of <0.05 was considered significant.

Results: No incidence of sternal dehiscence or postoperative complications was witnessed. Time to hemostasis, bone wax properties, number of dressing changes, sternal stability, pain, blood and blood products used, duration of ICU/hospital stay, reoperations, time taken to return back to normal day-to-day activities and to work, and subject satisfaction and QoL were comparable between Truwax^®^ and Ethicon^®^ bone wax groups.

Conclusion: Truwax^®^ and Ethicon^®^ bone waxes are safe and effective and provide sternal wound hemostasis in people undergoing sternotomy.

## Introduction

Sternotomy is considered the gold standard technique in cardiothoracic surgery, but well approximation is important to avoid postoperative bleeding, morbidity, and mortality [1]. Despite advancements in surgical techniques, sternal dehiscence, i.e., non-infectious sternum separation and/or movement, remains an important concern, affecting 0.4-1% of patients [2]. The mortality rate due to devascularization of the sternum is reported to be 25% [3]. The incidence of sternal wound infection is too frequent following sternotomy, ranging from 1% to 5%, that can lead to 10-30% death [4]. Unfavorable outcomes of sternotomy depend on the age of the patient, comorbidities, obesity, New York Hospital Association (NYHA) classification, surgical re-exploration, and lack of a standard technique or material to guarantee a low incidence of postoperative complications [5].

Bone wax is widely used over the sternal edges for achieving hemostasis [6]. Sterilized honeybee wax blended with paraffin is used as a barrier to control sternotomy bleeding [7]. The effectiveness of this hemostatic agent is highly debated as the incidence of infection, dehiscence, and inhibited osteogenesis are reported with bone wax use [7,8]. On the contrary, no clear indication of risk with bone wax use and effective hemostatic action were observed in other studies [9,10]. Additionally, there is a paucity of evidence regarding the comparative outcomes of two commonly used brands of bone wax for sternal wound hemostasis in subjects undergoing surgical procedures by sternotomy. Therefore, the present study compared the clinical equivalence of Truwax® (Healthium Medtech Limited, Bengaluru, India) with Ethicon® (Johnson & Johnson, New Brunswick, New Jersey, United States) bone wax, based on the incidence of sternal dehiscence and other postoperative complications within 26 weeks of median sternotomy closure.

## Materials and methods

Study design

The primary objective of this prospective (May 2022-April 2023), parallel-group, two-arm, randomized, single-blind, multicenter study was the comparison of sternal dehiscence rate within 26 weeks of the median sternotomy closure after applying Truwax® or Ethicon® bone wax. Secondary objectives were to elucidate the effect of two hemostats on average time to sternal hemostasis, overall intraoperative handling assessment of bone wax, effect of two hemostats on surgical site bleeding, postoperative wound healing, incisional pain post-sternotomy, and other postoperative complications of median sternotomy closure, viz., amount of postoperative drainage, bleeding, blood transfusion requirement, superficial and deep sternal wound infection, re-exploration (re-sternotomy) and all-cause mortality rates, subject satisfaction and health-related quality of life (QoL) post-sternotomy, time taken to return back to work and to normal day-to-day activities, material problems, and other adverse events (AEs) in both groups.

Ethics

The study was registered in the Clinical Trial Registry of India (CTRI) (registration number: CTRI/2022/03/041192 (registered on 17/03/2022)) and approved by the Ethics Committees of Mathikere Sampangi (MS) Ramaiah Medical College and Hospitals (date: 28/02/2022) and Ganesh Shankar Vidyarthi Memorial (GSVM) Medical College (approval number: EC/53/March/2022; date: 16/03/2022). Ethical principles of the Declaration of Helsinki Agreement, International Council for Harmonisation of Technical Requirements for Pharmaceuticals for Human Use (ICH) Guideline for Good Clinical Practice (GCP) E6 R2, European Standard (EN) International Organisation for Standardisation (ISO) 14155:2020, Indian Medical Devices Rules (MDR) 2017, MDR (EU) 2017/745, New Drugs and CT Rules 2019, and Consolidated Standards of Reporting Trials (CONSORT) were followed for conducting and reporting the study.

Study participants

Male or female adults (18-70 years) scheduled for sternotomy for surgical procedures on the heart, great vessels, and mediastinal lesions were included in the study after receiving written informed consent in the vernacular language prior to performing any study-specific procedures.

Participants with American Society of Anesthesiologists (ASA) grade 5 or hemoglobin (Hb) A1C of >10 or not requiring usage of bone wax to stop bleeding from the bone exposed by the sternotomy were excluded. Moreover, participants with a history of median sternotomy or bleeding disorders, or allergy to bee wax or similar products, or active infection at or around the skin incision site were excluded. Participants with a mental disorder/learning disability/language barrier or who received an experimental drug/device within 30 days prior to the study or pregnant or lactating female participants were also excluded. In addition to these, other criteria for exclusion were participation in another cardiovascular or similar study, or unlikely to comply with study procedure, or unlikely to complete the scheduled visits, or direct involvement in this study or other studies of the investigators or the institutions.

Study setting

The study was conducted at the Department of Cardiothoracic Surgery in MS Ramaiah Medical College and GSVM Medical College of India.

Intervention

Truwax® and Ethicon® bone waxes are a sterile mixture of bee wax, paraffin wax, and isopropyl palmitate. Both bone waxes are indicated to be used for the control of bleeding from bone surfaces; however, they are contraindicated to be used where rapid osseous regeneration and fusion are desired.

Study procedure

The pre-surgery screening was performed (week 26 to day -1), and the subjects were enrolled to undergo median sternotomy on day 0 for surgical procedures on the heart, great vessels, and mediastinal lesions. The surgery was performed in theatre by the investigators with recognized expertise in sternotomy along with their team members using institutional and standard surgical protocol. Immediately after sternotomy, Truwax® or Ethicon® bone wax was spread by digital pressure on the sternal surfaces to prevent sternal bleeding. Chest drains were connected to underwater seal drainage systems and left with suction to collect drainage from the pleural cavity. The presternal fascia, muscle, and skin were closed as per the standard institutional protocol. Subjects were transferred to the intensive care unit (ICU) for observation and continued their medical flow as per the standard of care. Subjects having a good postoperative recovery, no discharge from the sternal wound, and a stable sternum were discharged. Post-surgical follow-ups include day 3, days 4-15 (discharge), weeks 4-6, week 12, and week 26.

Baseline characteristics

Subject demographics, lab investigations regarding Hb, platelet count, prothrombin time, activated partial thromboplastin time (aPTT), and international normalized ratio (INR), classifications of ASA and NYHA, the reason for undergoing sternotomy, and history of smoking, alcohol consumption, treatment/surgery, and cardiovascular disease in the family were recorded. In addition, a physical examination for any anomaly was performed.

Study outcomes

Primary Outcome

A proportion of subjects having sternal dehiscence within 26 weeks of the median sternotomy closure was evaluated based on clinical findings of a sternal click or evidence of sternal instability during coughing or respiration. Chest radiography was performed as per investigator's discretion.

Secondary Outcomes

During the surgery, the surgeon watched both sides of the sternum for hemostasis after the application of bone wax, and the time from the start of the application to hemostasis was recorded. In addition, bone wax properties such as easy applicability, adhesiveness, easy to be shaped, satisfaction with sternal bleeding control, and packaging integrity were rated by the surgeons on a Likert scale (1 point for "very unsatisfied" and 5 points for "very satisfied"). Bleeding during surgery was based on the universal definition for perioperative bleeding (UDPB). Blood and its products like packed red blood cells (PRBCs), fresh frozen plasma (FFP), concentrated erythrocytes, platelet concentrates (PLT), and electrolyte solutions used were also captured. Other standard perioperative details such as type of surgery (full sternotomy/mini-sternotomy), any abnormality in the sternum, whether osteoporotic sternum, number of grafts, length of surgery, sternotomy incision length, cardiopulmonary bypass time, amount of bone wax used, quantity of initially used bone wax removed from the area of application before approximating the sternum, description of the surgery, type of suture used for sternal closure, number of blood transfusion, antibiotic and thrombosis prophylaxis, and perioperative complications were also recorded.

Postoperative bleeding/oozing from the surgical site was clinically assessed, and the number of dressing changes was noted. The Sternal Instability Scale (0 is "clinically stable sternum" and 3 is "completely separated sternum") was used to assess the stability of the sternum [11]. Sternal bone instability was only confirmed by chest radiography at the investigator's discretion. Findings such as a mid-sternum line of lucency of >2 mm indicating sternum diastasis, any sternum wire displacement, and any obvious interruption/dislocation were recorded in case a chest radiograph was performed. Postoperative experience of pain at rest, during coughing, and on movement was assessed using the Visual Analogue Scale (VAS) with 0 corresponding to "no pain" and 100 to "worst pain." Other complications after sternotomy, viz., amount of postoperative drainage through mediastinal drains in the first 24 hours and till discharge, superficial and deep sternal wound infection, reoperations, mediastinitis, hemothorax, steel wire fracture, all-cause mortality rates, other complications of sternal closure, other bone wax-related complications, and blood and blood products used postoperatively, were recorded. Moreover, the duration of stay at the ICU and hospital and time taken to return back to work and to normal day-to-day activities were noted. Subject satisfaction and QoL were measured using the EuroQoL Five-Dimensional Three-Level (EQ-5D-3L) Questionnaire. The five dimensions of EQ-5D-3L comprised mobility, self-care, usual activities, pain or discomfort, and anxiety or depression, which were marked on three levels: no, some, and extreme problems. Similarly, EuroQoL-VAS (EQ-VAS) was used for the assessment of the subject's health on a 0-100 scale, where 0 is the worst imaginable health and 100 is the best imaginable health.

Any abnormal medical occurrence/disease/injury/clinical sign was considered as an AE. Serious AE was marked when a subject had any life-threatening condition, leading to hospitalization or death. Concomitant or prescribed medications were also documented.

Sample size

The postoperative sternal dehiscence rate was reported as 1.4% in patients without bone wax and 2.5% in patients with bone wax [12]. Therefore, the proportion of subjects with sternal dehiscence in the Ethicon® bone wax arm was assumed as 2.5%, i.e., π1=0.025. Further assuming a type I error as 5%, power as 80%, and a difference to be detected as 0.2% for the proportion of subjects having sternal dehiscence in the Truwax® arm (π_2_=2.7%, 0.027), with 10% margin of non-inferiority, the sample size of 38 in each arm was required. The total sample size requirement was 76. The required sample size was increased to 92 (46 subjects in each arm) after considering 20% dropout and post-randomization exclusion. Two centers were involved in the study and enrolled a proposed sample of up to 46 subjects, with 23 subjects block randomized to the Truwax® arm and 23 subjects to the Ethicon® bone wax arm. The sample size calculation formula was as follows: π_1_-π_2_>δ n_i_=(Z_α_+Z_β_)^2^(π_1_(1-π_2_)+π_2_(1-π_2_))/(π_1_-π_2_ -δ)^2^ (two-sample parallel non-inferiority). Here, n_i_ is the sample size required in each group; Z_α_, the conventional multiplier for alpha; Z_β_, the conventional multiplier for power; π_1_, the proportion of patients having sternal dehiscence in the Ethicon® bone wax arm; π_2_, the proportion of patients having sternal dehiscence in the Truwax® arm; δ, the margin of non-inferiority difference; and π_1_-π_2_, the size of difference of clinical importance.

Randomization and blinding

Two random lists of size n=46 (23 vs. 23) were generated by Random Allocation Software (V 1.0) using block sizes of 4, 6, or 8. Block randomization of variable block length as per study sites was performed, and the randomization codes were issued to the sites in sequentially numbered opaque sealed envelopes. Subjects were allocated randomly to one of the two treatment arms (Truwax® or Ethicon® bone wax) just before the surgical procedure. The detail of the treatment arm was blinded to the subjects only.

Statistical analysis

Statistical analysis of primary and secondary outcomes was performed using IBM SPSS Statistics for Windows, Version 28.0 (Released 2021; IBM Corp., Armonk, New York, United States) in a per-protocol (PP) analysis set, consisting of all randomized subjects who completed the study till 26 weeks. Continuous variables were represented as mean±SD and categorical variables as numbers (n) and percentages (%). For comparisons of continuous variables, the t-test or Mann-Whitney U test (depending on data distribution) was used, and for categorical variables, the chi-squared test was used. A probability of less than 0.05 was considered significant.

## Results

**Figure 1:**
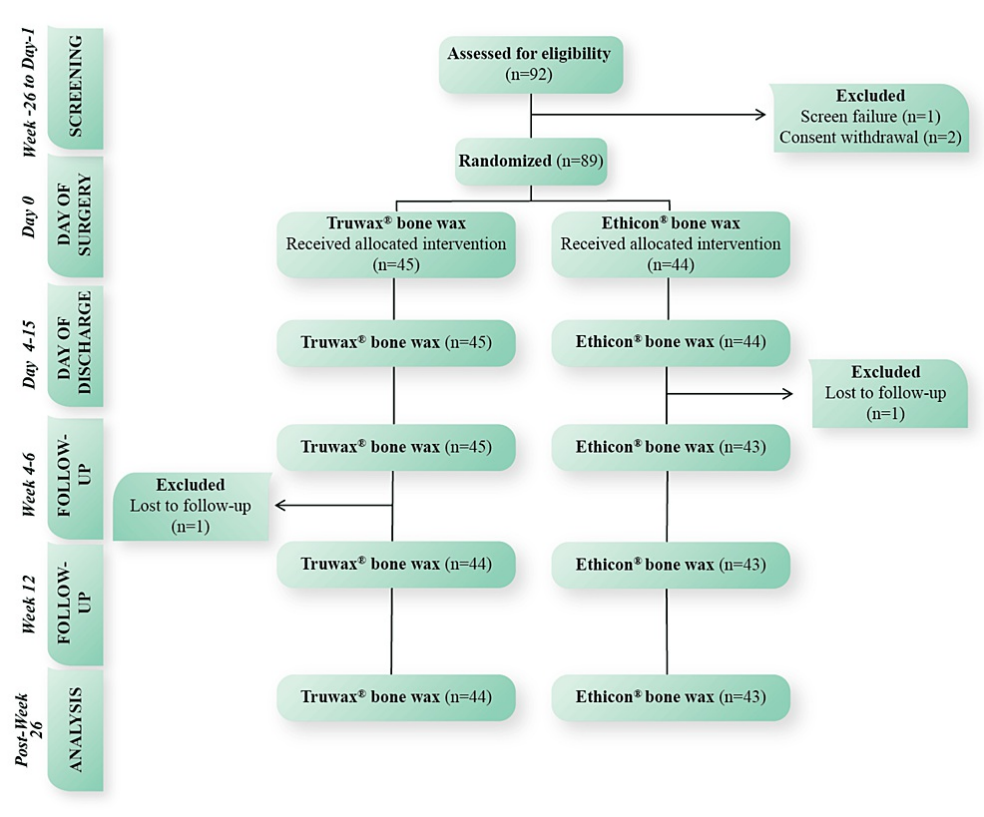
CONSORT diagram of study participants n: number of subjects; CONSORT: Consolidated Standards of Reporting Trials

A total of 92 participants were screened (May 2022-September 2023); 89 met the eligibility criteria and were randomized to either group (Figure 1).

One subject randomized to the Truwax® bone wax group and one to the Ethicon® bone wax group were excluded after being lost to follow-up at week 12 and weeks 4-6, respectively. Therefore, the PP analysis set had 87 randomized subjects (Truwax® bone wax (n=44) and Ethicon® bone wax (n=43)) having complete data of primary effectiveness parameters and had no major protocol deviations that could impact the primary outcome of this study.

Baseline characteristics

The mean age of the study participants was 53.2±10.5 years in the Truwax® bone wax group and 52.7±12.6 years in the Ethicon® bone wax group (p=0.08). Twenty-four subjects in both Truwax® bone wax (54.5%) and Ethicon® bone wax (55.8%) groups were Indians, while others were non-Indian Asians (p=0.91). Male dominance was prevalent in the gender distribution of the study participants, with a significant difference between Truwax® and Ethicon® bone wax groups (81.8% vs. 58.1%, p=0.02). The occupation of the majority of subjects in both groups involved a mild strenuous job (65.9% vs. 53.4%, p=0.53). Subject characteristics, vital signs, findings of lab investigations, and ASA and NYHA classifications were similar (Table 1).

**Table 1:** Preoperative profile of study participants Value representation as mean±SD or n (%) aPTT: activated partial thromboplastin time; ASA: American Society of Anesthesiologists; BMI: body mass index; Hb: hemoglobin; INR: international normalized ratio; NYHA: New York Heart Association; SD: standard deviation; n: number of subjects

Subject characteristics	Truwax^®^ bone wax (n=44)	Ethicon^®^ bone wax (n=43)	P-value
Demographics			
Weight (kg)	66.9±9.6	62.4±9.6	0.56
Height (cm)	164.6±8.1	160.9±9.1	0.32
BMI (kg/m^2^)	24.7±3.5	24.2±3.4	0.45
Vital signs			
Pulse rate (beats per minute)	82.3±10.9	82.1±9.5	0.55
Respiratory rate (per minute)	18±2.5	17.6±2.5	0.53
Systolic blood pressure (mmHg)	121.5±7.2	123±11.4	0.31
Diastolic blood pressure (mmHg)	80.3±6.3	77.9±7.8	0.09
Lab investigations			
Hb (g/dL)	12.6±1.7	12.5±1.9	0.62
Platelet count (x10³/microL)	2.2±0.7 (n=43)	2.1±0.8 (n=41)	0.58
Prothrombin time (seconds)	16±7.4 (n=41)	18±10 (n=39)	0.31
aPTT (seconds)	24.1±10.5 (n=25)	25.1±10.6 (n=21)	0.76
INR	1.3±0.5 (n=42)	1.4±0.7 (n=41)	0.15
ASA grade			
ASA I	15 (34.1)	17 (39.5)	0.31
ASA II	12 (27.3)	6 (14)	
ASA III	17 (38.6)	20 (46.5)	
NYHA classification			
NYHA I	13 (29.5)	19 (44.2)	0.29
NYHA II	25 (56.8)	21 (48.8)	
NYHA III	6 (13.6)	3 (7)	

History of alcohol consumption (9.1% vs. 9.3%, p=0.97) and smoking (9.1% vs. 7%, p=0.72) were comparable between the groups. Only 2.3% and 7% of subjects in Truwax® and Ethicon® bone wax groups, respectively, had a family history of cardiovascular disease (p=0.30). All subjects had medical/surgery history related to the cardiovascular system. Physical examination also recorded abnormal cardiovascular system in all subjects, requiring open surgical procedures on the heart.

Primary endpoint analysis

The incidence of sternal dehiscence within 26 weeks of the median sternotomy closure was not recorded in this study. Although a significant difference (p<0.05) was noted in gender and usual activities of EQ-5D at the screening visit between the groups, this did not influence the primary outcome, as the incidence of sternal dehiscence is nil in both groups.

Secondary endpoint analysis

Intraoperative Profile

Sternotomy was performed in all subjects, and none were found to have any sternum abnormality or osteoporotic sternum. All subjects had received intraoperative prophylactic systemic antibiotic therapy. Piperacillin with tazobactam (40.9% vs. 39.5%) and meropenem (52.3% vs. 51.2%) were the most frequently given antibiotics in Truwax® and Ethicon® bone wax groups (p=0.67). The majority of subjects of both groups underwent coronary artery bypass graft surgery (70.5% vs. 69.8%), followed by valve replacement (25% vs. 20.9%); coronary artery bypass graft surgery with valve replacement, atrial mass excision, heart transplant, and sternotomy with pericardiocentesis were also performed (p=0.62). Graft or prosthesis in 97.7% and 95.3% of subjects (p=0.54) and cardiopulmonary bypass in 20.5% and 14% of subjects (p=0.42) of the Truwax® and Ethicon® bone wax groups, respectively, were used. The duration of cardiopulmonary bypass, time from the start of application of bone wax to hemostasis, and other intraoperative details are provided in Table 2.

**Table 2:** Intraoperative and postoperative profile of study participants Value representation as mean±SD or n (%) aPTT: activated partial thromboplastin time; Hb: hemoglobin; ICU: intensive care unit; INR: international normalized ratio; NYHA: New York Heart Association; SD: standard deviation; UDPB: universal definition for perioperative bleeding; n: number of subjects

Subject profile	Truwax^® ^bone wax (n=44)	Ethicon^®^ bone wax (n=43)	P-value
Intraoperative			
Sternotomy incision length (cm)	17.1±0.8	17.2±1	0.72
Length of surgery (hours)	4.4±1.9	4.4±2.2	0.21
Time from the start of application of bone wax to hemostasis (seconds)	36.1±24.2	35.4±24.4	0.47
Quantity of bone wax used (gm)	2.4±0.6	2.4±0.7	0.83
Mechanical ventilation time (minutes)	372±88.4	384.4±107.7	0.90
Cardiopulmonary time (minutes)	61.8±21.3 (n=9)	41.7±9.8 (n=6)	0.08
Bleeding grade			
UDPB class 0	32 (72.7)	33 (76.7)	0.12
UDPB class 1	4 (9.1)	0	
UDPB class 2	8 (18.2)	10 (23.3)	
Chest tube drainage volume at 24 hours (ml)	506.4±131.4	565.6±215.3	0.34
Postoperative			
Hb (g/dL)	13.2±1.6 (n=28)	13.3±1 (n=27)	0.24
Prothrombin time (seconds)	14.4±11.1 (n=21)	16.3±13.8 (n=22)	0.27
aPTT (seconds)	3.8±0.5 (n=4)	4.6±2.6 (n=5)	0.08
INR	1.8±0.5 (n=22)	1.7±0.4 (n=20)	0.13
Bleeding/oozing from the surgical site			
Day 3	18 (40.9)	17 (39.5)	0.90
Days 4-15	5 (11.3)	9 (20.9)	0.23
Number of dressing changes			
72 hours post-surgery	1.9±1.3	2.1±1.4	0.79
After day 3	1.1±1.2	1.1±1.2	0.53
Chest tube drainage volume till discharge (ml)	696.1±305.3	778.3±268.2	0.69
Duration of drain (days)	3.5±2.4	4.1±2.7	0.38
Duration of ICU stay (days)	3.3±1.7	3.7±2.4	0.17
Duration of hospital stay (days)	8.1±2.5	9.2±3.7	0.09
Time taken to return to normal day-to-day activities (days)	28.1±9.3	29.2±9.8	0.59
Time taken to return to work (days)	59.4±14.5	62.2±9.4	0.37

**Figure 2:**
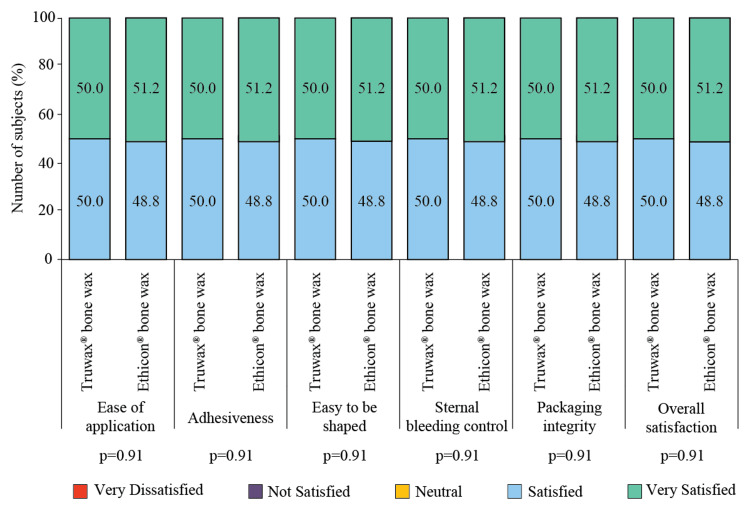
Intraoperative rating of Truwax® bone wax (n=44) and Ethicon® bone wax (n=43) properties

The sternum was closed with a figure-of-8 configuration technique using a steel suture. The perspective of the surgeons for Truwax® and Ethicon® bone wax was "very satisfied" or "satisfied" (Figure 2).

In Truwax® and Ethicon® bone wax groups, heparin (50% vs. 51.2%) and enoxaparin (43.2% vs. 39.5%) were used as the thrombosis prophylaxis (p=0.67). Re-bleeding occurred in 4.5% and 7% of subjects of the Truwax® and Ethicon® bone wax groups, respectively (p=0.63), and the same bone wax was reapplied to stop the bleeding. Insignificant perioperative bleeding was more prevalent in both Truwax® bone wax (72.7%) and Ethicon® bone wax (76.7%) groups (p=0.12); however, mild and moderate UDPB class was also evident (Table 2). Perioperative blood and blood products were used in 97.7% and 95.3% of subjects of the Truwax® and Ethicon® bone wax groups, respectively (p=0.29), that include whole blood, PRBCs, FFP, PLT, and electrolyte solution. Good outcome of surgery was recorded in both groups as no perioperative or bone wax-related complications were noted.

Postoperative Profile

Reduced requirement of blood and blood products was evident postoperatively, as only PRBCs (1 unit) were transfused to 34.1% and 27.9% of subjects of the Truwax® and Ethicon® bone wax groups, respectively (p=0.53), at days 4-15. The number of dressing changes was also decreased in both groups (Table 2). During the surgery, drain was administered in all subjects and removed by days 4-15. Chest tube drainage volume till discharge was comparable between Truwax® and Ethicon® bone wax groups (Table 2). Post-sternotomy complications including all-cause mortality, superficial and deep sternal wound infection, reoperations, mediastinitis, hemothorax, steel wire fracture, other complications of sternal closure, and other bone wax-related complications were not observed.

**Figure 3:**
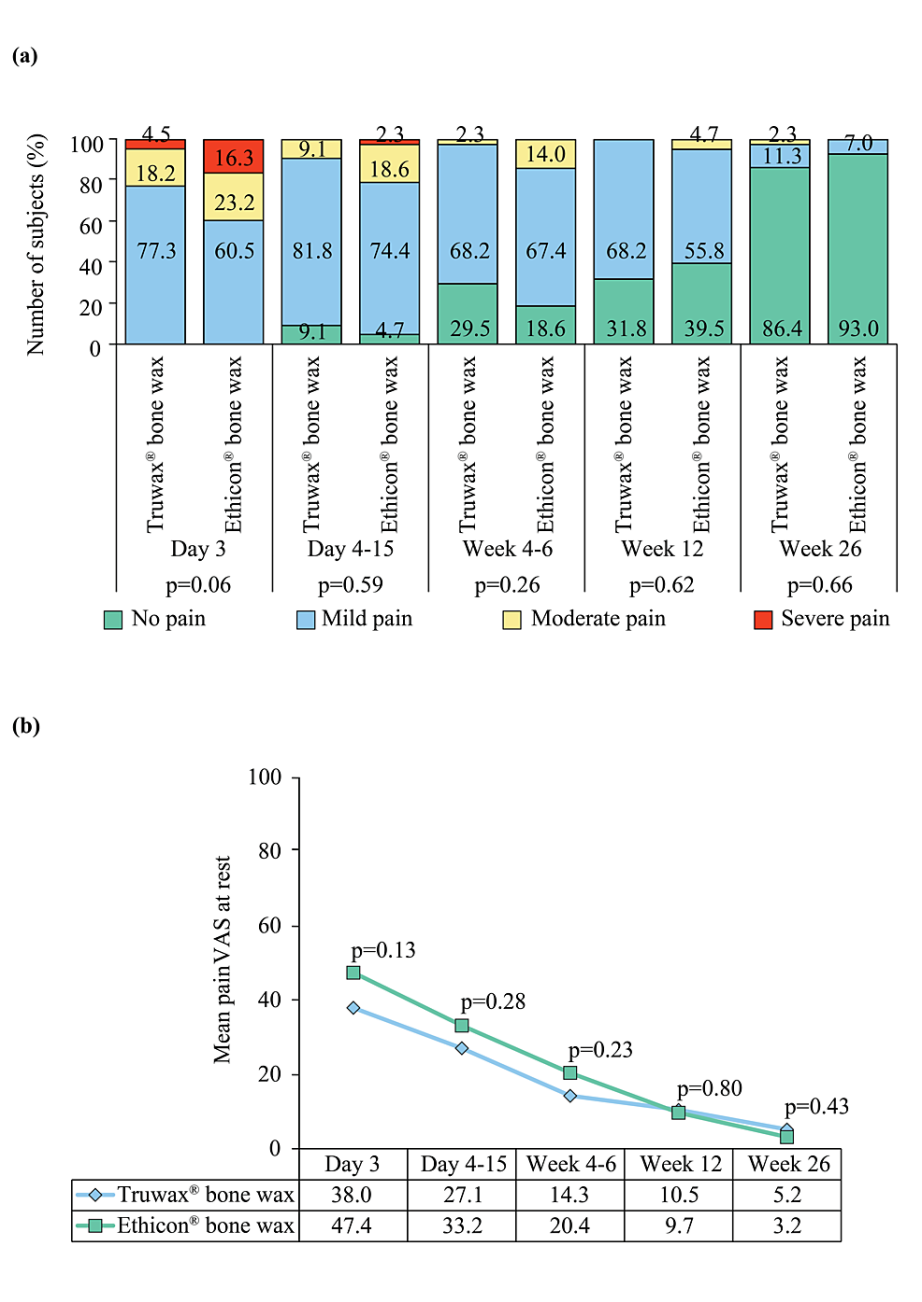
Proportion of wound pain at rest (a) and mean wound pain at rest (b) in Truwax® bone wax (n=44) and Ethicon® bone wax (n=43) groups VAS: Visual Analogue Scale

Mild, moderate, or severe cough was present in all subjects postoperatively. However, the frequency of cough reduced, and at the last follow-up, 4.5% and 2.3% of subjects of the Truwax® and Ethicon® bone wax groups, respectively, had a mild cough, and only 2.3% of subjects of the Truwax® bone wax group had a moderate cough (p=0.99). In Truwax® and Ethicon® bone wax groups, mild and moderate pain during coughing were experienced by 9.1% vs. 2.3% and 4.5% vs. 4.7% of subjects, respectively, on day 3 (p=0.06) and by 2.3% vs. 4.7% and 4.5% vs. 2.3% of subjects, respectively, on days 4-15; severe pain was also recorded in 2.3% of subject of the Ethicon® bone wax group on days 4-15 (p=0.32). After discharge, severe pain during coughing was noted in none of the subjects. Pain during coughing at weeks 4-6 (mild: 2.3% vs. 0% and moderate: 0% vs. 2.3%, p=0.16), week 12 (mild: 4.5% vs. 2.3% and moderate: 4.5% vs. 2.3%, p=0.31), and week 26 (mild: 6.8% vs. 2.3%, p=0.26) were comparable between the Truwax® and Ethicon® bone wax groups. The mean VAS of subjects who had cough was comparable at day 3 (43.3±19.7 vs. 73.5±26.1, p=0.79), days 4-15 (52.7±25.4 vs. 53±27, p=0.99), weeks 4-6 (20 vs. 56, p=0.32), week 12 (41.3±19.4 vs. 42.5±27.6, p=0.88), and week 26 (12.7±10 vs. 10, p=0.66) between the groups. After recovering from anesthesia in Truwax® and Ethicon® bone wax groups, 18.2% and 23.3%, respectively, complained of severe pain and 25% and 25.6%, respectively, of moderate pain, while 56.8% and 51.2% had mild pain (p=0.82). The mean VAS of wound pain after surgery was recorded as 50.4±24.6 in the Truwax® and 53.1±25.5 in the Ethicon® bone wax group, and the result was comparable (p=0.62). The grade and mean VAS of wound pain at rest at all postoperative visits were comparable between the groups (Figure 3a, 3b).

**Figure 4:**
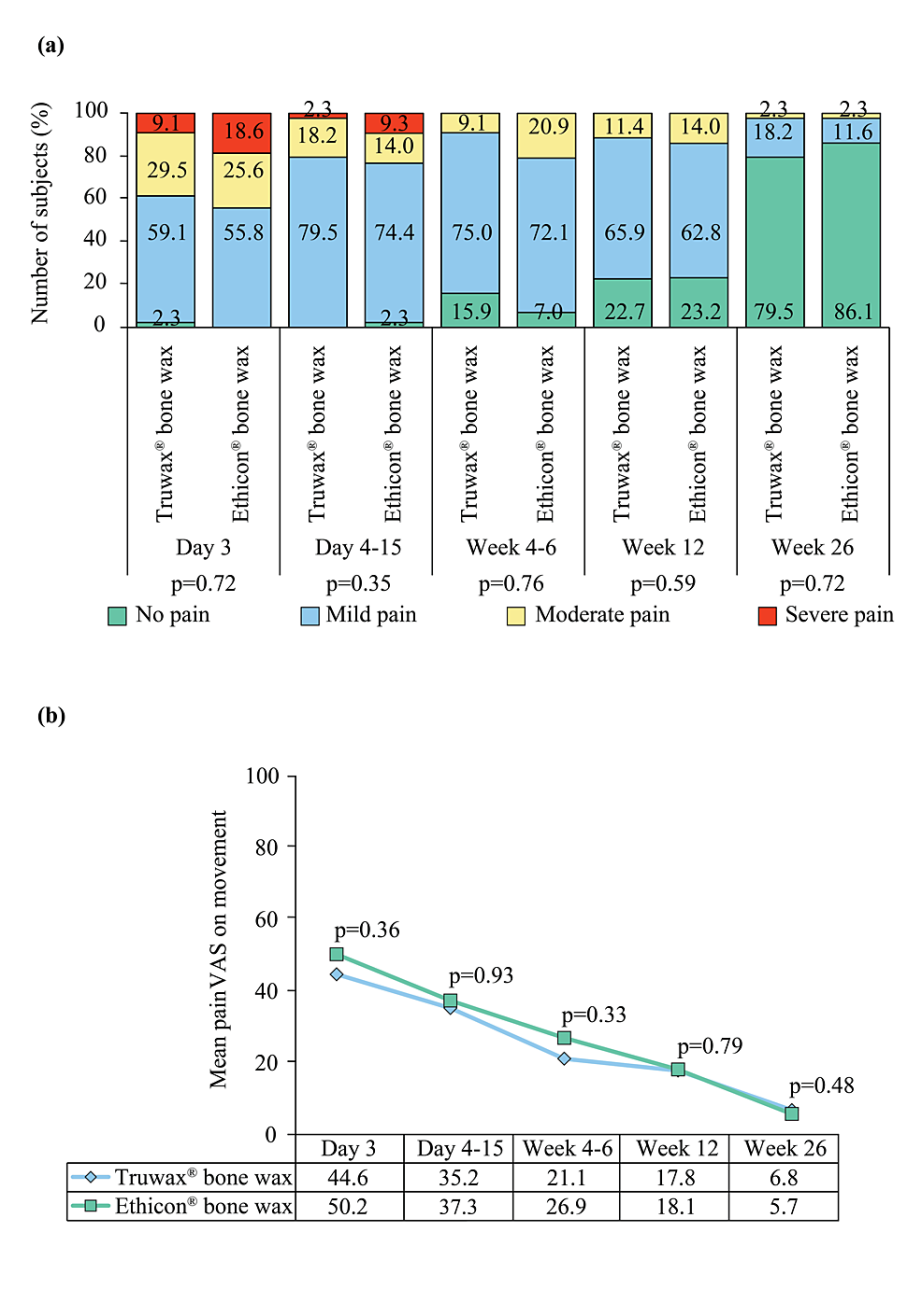
Proportion of wound pain on movement (a) and mean wound pain on movement (b) in Truwax® bone wax (n=44) and Ethicon® bone wax (n=43) groups VAS: Visual Analogue Scale

The grade and mean VAS of wound pain on movement at all postoperative visits were also comparable between the groups (Figure 4a, 4b).

**Figure 5:**
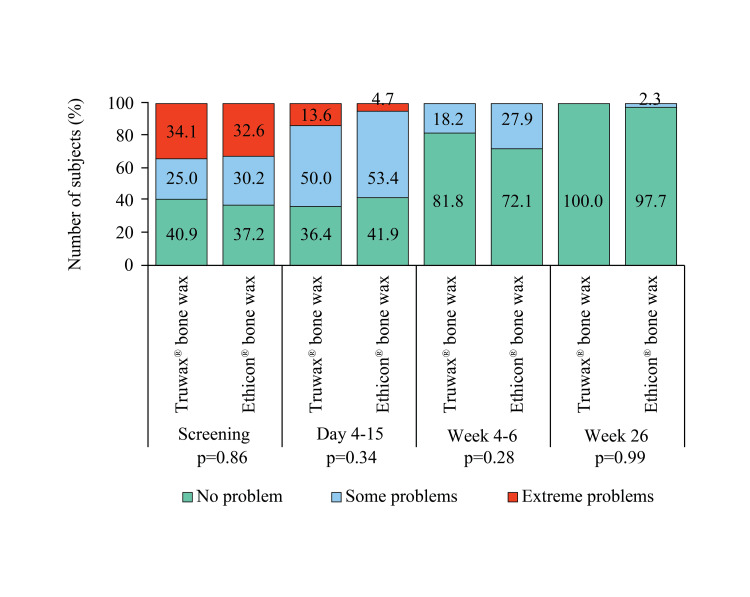
EuroQoL assessment of mobility in Truwax® bone wax (n=44) and Ethicon® bone wax (n=43) groups

A clinically stable sternum was found in all subjects. However, chest radiograph was carried out in Truwax® and Ethicon® bone wax groups at day 3 (93.2% vs. 88.4%, p=0.44), days 4-15 (65.9% vs. 65.1%, p=0.82), weeks 4-6 (13.6% vs. 18.6%, p=0.37), and week 26 (2.3% vs. 4.7%, p=0.31). The findings revealed a mid-sternum line of lucency of ≤2 mm, no displacement of any sternum wire, or any obvious interruption or dislocation in these subjects. The duration of ICU and hospital stay were comparable between the groups (Table 2). Readmission was required in one subject of the Truwax® bone wax group, due to increased INR (>8); the event was reported as serious AE (not related to the study device). However, the subject continued in the study after discharge. The time taken to return to normal day-to-day activities and to work was comparable between the groups (Table 2). Global assessment of EQ-5D showed postoperative improvement in the mobility of subjects in both Truwax® and Ethicon® bone wax groups (Figure 5).

**Figure 6:**
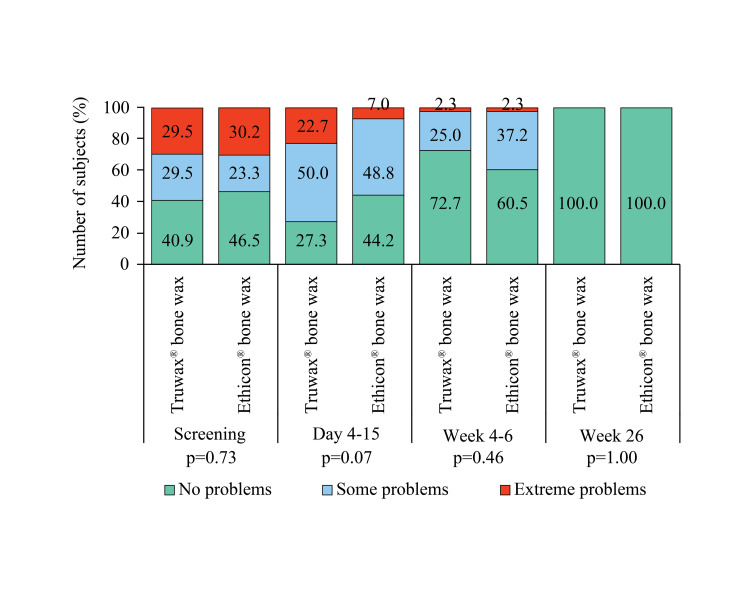
EuroQoL assessment of self-care in Truwax® bone wax (n=44) and Ethicon® bone wax (n=43) groups

Moreover, no problem in taking self-care at week 26 was reported by all subjects of both groups (Figure 6).

**Figure 7:**
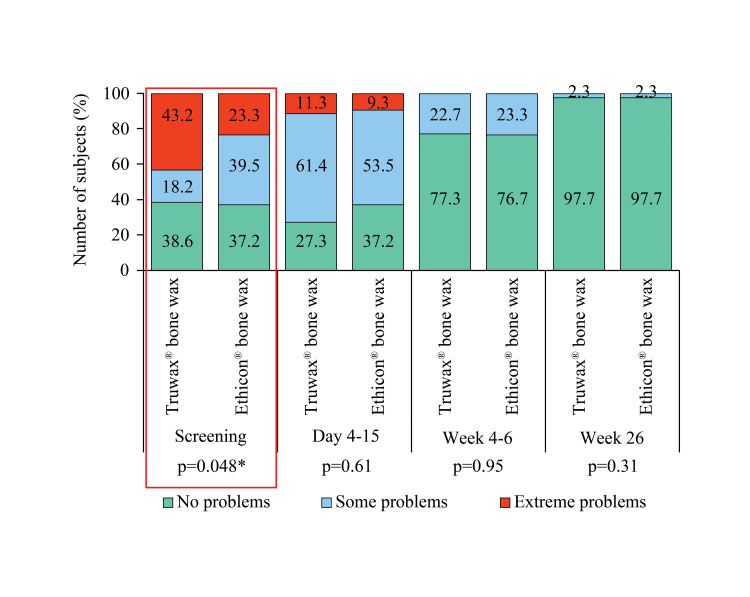
EuroQoL assessment of usual activities in Truwax® bone wax (n=44) and Ethicon® bone wax (n=43) groups *p<0.05

A significant difference (p<0.05) in doing usual activities before surgery was noted between the groups. However, the result was comparable between Truwax® and Ethicon® bone wax groups at days 4-15, weeks 4-6, and week 26 (Figure 7).

**Figure 8:**
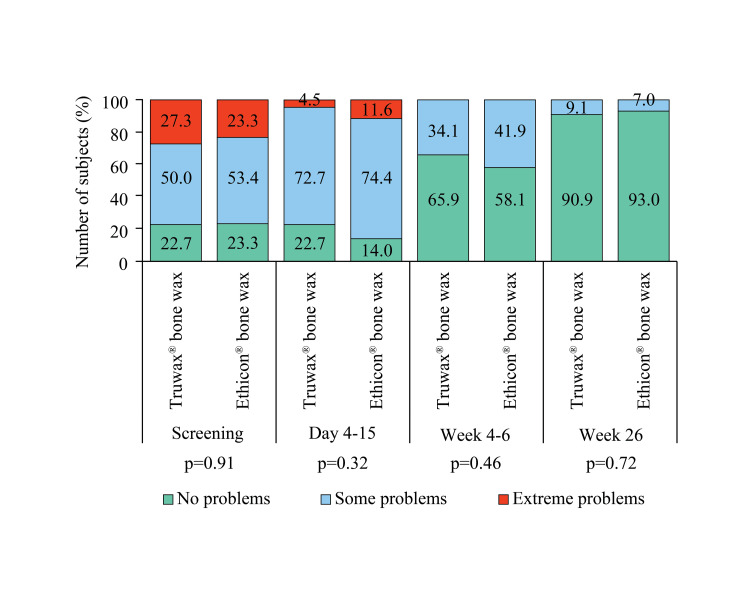
EuroQoL assessment of pain/discomfort in Truwax® bone wax (n=44) and Ethicon® bone wax (n=43) groups

No pain or discomfort was reported by the majority of subjects of both groups at the last follow-up (Figure 8).

**Figure 9:**
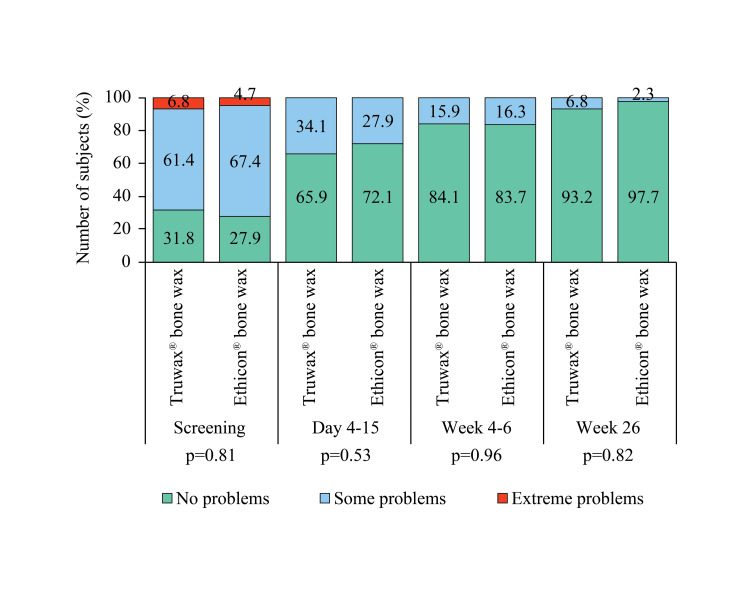
EuroQoL assessment of anxiety/depression in Truwax® bone wax (n=44) and Ethicon® bone wax (n=43) groups

Improvement in anxiety or depression with each follow-up visit was also recorded (Figure 9).

**Figure 10:**
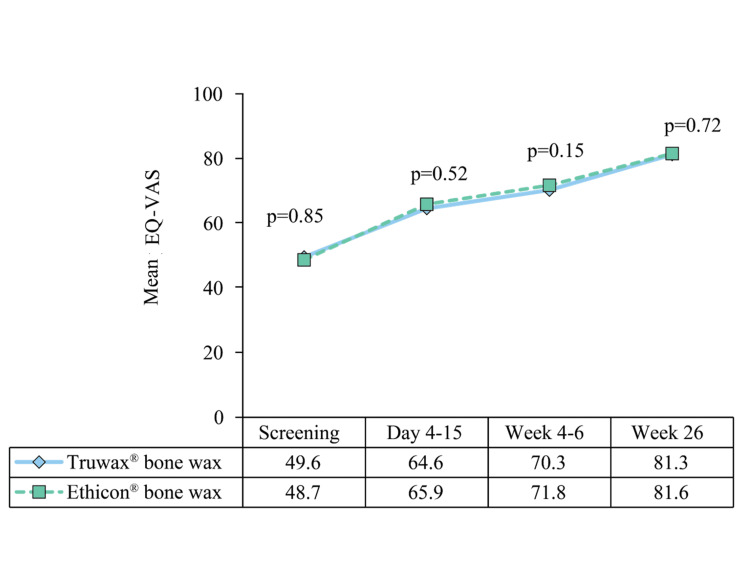
EQ-VAS assessment in Truwax® bone wax (n=44) and Ethicon® bone wax (n=43) groups

In addition, findings of EQ-VAS in both groups demonstrated a similar improvement in subjects' QoL following median sternotomy (Figure 10).

In both Truwax® and Ethicon® bone wax groups, anxiety (29.5% vs. 18.6%) and gastrointestinal AEs including gastroesophageal reflux disease (54.5% vs. 62.8%), gastritis (15.9% vs. 23.3%), and constipation (29.5% vs. 27.9%) were the most common AEs. All AEs were mild and not related to the study devices. Majorly used concomitant or prescribed medications during the study are summarized in Table 3.

**Table 3:** Concomitant or prescribed medications Value representation as n (%) N: number of subjects

Concomitant/prescribed medications	Truwax^®^ bone wax (n=44)	Ethicon^®^ bone wax (n=43)
Analgesics		
Paracetamol	20 (45.5)	19 (44.2)
Tapentadol	12 (27.3)	7 (16.3)
Antibiotics		
Faropenem	21 (47.7)	19 (44.2)
Levofloxacin	23 (52.3)	20 (46.5)
Linezolid	5 (11.3)	9 (20.9)
Gastrointestinal		
Pantoprazole	21 (47.7)	19 (44.2)
Domperidone+rabeprazole	15 (34.1)	13 (30.2)
Domperidone+pantoprazole	13 (29.5)	10 (23.3)
Others		
Aspirin	34 (77.3)	27 (62.8)
Furosemide+spiranolactone	20 (45.5)	20 (46.5)
Metoprolol	23 (52.3)	17 (39.5)
Atorvastatin	26 (59.1)	25 (58.1)
Clopidogrel	18 (40.9)	18 (41.9)
Torsemide	18 (40.9)	16 (37.2)
Nicorandil	21 (47.7)	16 (37.2)
Cremaffin	21 (47.7)	19 (44.2)
Warfarin	10 (22.7)	15 (34.9)
Insulin	17 (38.6)	11 (25.6)

## Discussion

Sternotomy is a classical technique for the surgical repair of congenital heart defects, the success of which leads to minimized post-surgery morbidity and increased survival. Favorable outcomes can be augmented with the elimination of risk factors, choice of surgical technique, cardiopulmonary bypass technology, antibiotic prophylaxis, and postoperative care [13]. In the present 26 weeks of study, the clinical equivalence of Truwax® and Ethicon® bone waxes for sternal wound hemostasis was compared. Bone wax is commonly used in thoracic surgeries for providing an impenetrable mechanical barrier to blood flow from the transected vessels [14,15] as postoperative bleeding may develop wound infection [6]. Investigators of the present study were "very satisfied" or "satisfied" with the ease of application, adhesiveness, ease to be shaped, control of sternal bleeding, packaging integrity, and overall satisfaction of the Truwax® and Ethicon® bone waxes. Sternotomy was performed in all subjects requiring open surgical procedures on the heart. Steel suture was used for sternum closure using the figure-of-8 configuration technique, and all subjects have received antibiotic prophylaxis. Despite advances in surgical techniques and postoperative observation, there is still a 0.8-1.5% probability of sternal dehiscence and mediastinitis [6]. Deep sternal wound complications often require long operations and re-exploration and are associated with postoperative bleeding [16]. A prospective observational study reported 2.5% cases of sternal dehiscence with the use of bone wax [12]. Within three months of median sternotomy, 1/25 cases of sternal dehiscence and 1/25 superficial wound infections were also recorded in subjects who received bone wax for sternal closure concomitant with electrocauterization [8]. Unlikely, no evidence of deep sternal infection with the application of bone wax was found in a previous study [9]. Similarly, the present study recorded no postoperative sternal dehiscence, mediastinitis, and superficial and deep sternal wound infection after using bone wax. In addition, reoperation after sternal closure was not required in subjects undergoing sternotomy.

Post-sternotomy bleeding occurs extensively, requiring 20% transfusion of blood products worldwide, and may result in increased hospitalization and mortality and may impact patient outcomes. The UDPB class is proportionate to mortality independent of cardiopulmonary bypass duration [17]. However, active tube clearance of chest tubes reduces re-exploration and blood complications [18]. Although UDPB classes 1 and 2 were noted in some subjects of Truwax® and Ethicon® bone wax groups, insignificant or UDPB class 0 bleeding was more prevalent in both arms. Bleeding/oozing from the surgical site has decreased from day 3 to days 4-15 and so has the number of dressing changes. Consequently, postoperative use of blood products was decreased on these visits.

Coughing increases intrathoracic pressure and exerts excessive tension on the sternum in turn increasing the risk for sternal instability [19]. The latter is characterized by sternal clicking, difficulty in mobility, and pain with no sign of healing that hinders patient QoL and increases the risk of mediastinitis [20]. Mild/moderate coughing was recorded in both Truwax® and Ethicon® bone wax groups, and towards the end of the study, only a few subjects had a cough. The pain experienced during coughing was not too intense and only of mild type as found at the last follow-up. Moreover, severe pain was recorded neither at rest nor on movement after discharge from the hospital. Additionally, all subjects had clinically stable sternum without any incidence of sternum wire displacement, wire fracture, or any obvious interruption/dislocation.

Besides functional recovery, minimal pain, improved QoL, and patient morbidity are assessed based on ICU/hospital stay as a prolonged stay may relate to hemodynamic instability or reoperations [13]. The mean stay at ICU (~3 days) and hospital (~8-9 days) was similar between Truwax® and Ethicon® bone wax groups of the present study, and readmission of one subject of the Truwax® group did not impact the study outcomes. A study by Zheng et al. reported higher responsiveness of the EQ-5D-3L Questionnaire among patients with coronary heart disease [21]. The present study also offers evidences of improved mobility, pain/discomfort, anxiety/depression, and ability to self-care and to perform usual activities with each post-sternotomy follow-up. In addition, the time duration for return to work was similar between the study groups. Return to day-to-day activities was ~1 day earlier in the Truwax® bone wax group than the Ethicon® bone wax group. Gastrointestinal complications following cardiac surgery are one of the reasons for mortality. Many risk factors including hypoperfusion of abdominal viscera after cardiopulmonary bypass increase susceptibility to develop these complications [22]. Gastrointestinal AEs are frequent in the present study; however, the events did not require hospitalization and got better after treatment. Moreover, an absolute survival rate was recorded. 

The multifaceted strengths of this study are that it may be the only study to evaluate the effectiveness of two sterile bone wax in sternotomy and assess wide possibilities of post-sternotomy complications in the form of primary and secondary endpoints. The study outcomes will help to validate the clinical use of Truwax® bone wax in a larger population. Meanwhile, the only limitation is the perspective of the surgeons on bone wax properties. As this is a single-blind study, the surgeons could not be blinded, raising the chances of potential bias in the overall rating of the bone wax properties.

## Conclusions

The present findings demonstrated no incidence of sternal dehiscence within 26 weeks of median sternotomy closure using Truwax® and Ethicon® bone waxes. In addition, comparable findings of average time to hemostasis, bone wax properties, number of dressing changes, sternal bone instability, pain, perioperative/postoperative complications, blood and blood products used, duration of ICU/hospital stay, reoperation, time taken to return back to day-to-day activities and work, and subject satisfaction and QoL indicated the clinical equivalence of Truwax® and Ethicon® bone waxes. Therefore, Truwax® and Ethicon® bone waxes are safe and effective and provide sternal wound hemostasis in surgical procedures by sternotomy closure.
